# Timeliness of Communication: An Audit of Clinic Documentation and Outpatient Correspondence Within Community Mental Health Teams

**DOI:** 10.1192/bjo.2026.11712

**Published:** 2026-06-30

**Authors:** Abdul Manan Gul

**Affiliations:** Northamptonshire Healthcare NHS Foundation trust (NHFT), Northampton, United Kingdom

## Abstract

**Aims:**

Effective communication between secondary and primary care is fundamental to patient safety and continuity of care. While the GMC and RCPsych emphasize high-quality correspondence, there is a lack of strictly mandated national timeframes for non-urgent outpatient letters. In line with the NHS Standard Contract, our Trust (NHFT) utilizes a 24-hour benchmark for clinic entry completion and a 7-day standard for issuing GP letters. This audit aimed to evaluate compliance with these standards across the North and South Community Mental Health Teams (CMHTs). The Set standard was that 100% of clinic outcomes and clinic letters should be completed within Trust’s time standards of 24 hours and 7 days, respectively.

**Methods:**

A retrospective audit was conducted over a six-week period (24 March–2 May 2025). Data was collected from SystmOne (Trust’s Electronic record system) for 176 clinics across various localities.

Primarily, the timeframe within which clinic outcomes were completed was evaluated against standards. Secondarily, the same was done for Clinic letter completion timeframe. The final compliance rates were compared between the North and South teams, to assess for any variability.

**Results:**

Of the 176 clinics audited (North: 62; South: 114), overall compliance was high, although it was below the target of 100%.

- **Clinic Entries (<24 hours):** South CMHTs achieved 99% compliance, while North CMHTs achieved 94%.
- **Clinic Letters (<7 days):** South CMHTs demonstrated excellent timeliness at 99%. However, North, 92% of letters met the 7-day target. In the North locality, 8% of letters were overdue or missing, with some delays extending up to 31 days. Conversely, the South locality had only one missing letter and one overdue letter, representing less than 1% of the sample.

**Conclusion:**

The overall compliance was below set standards. Identified factors included missing letters and significant delays (>30 days). The clinicians involved were not aware of the undergoing audit.

In addition to the short duration, the audit didn’t account for the total number of appointments, patient non-attendance and administrative capacity.

We recommend that the findings are presented to regional academic teaching program, a survey to identify administrative capacity issues, a longer re-audit duration of 12 weeks, inclusion of the newly implemented Local Area Partnership (LAP) transformation model, as well as addressing the identified limitations. Going a further step, we recommend the addition of consenting patients as recipients according to the new clinic-letter standard.

